# General Nursing Council for Ireland

**Published:** 1921-03-12

**Authors:** 


### General Nursing Council for Ireland.

The fourth meeting of this Council was held on February 25. There were present Sir Edward Coey Bigger, M.D. (Chairman), Miss Huxley (Vice-Chairman), Lady Kenmare, Sir Arthur Chance, F.R.C.S.I., Sir William Taylor, C.B., F.R.C.S.I., Mr. P. T. O'Sullivan, M.D., Miss O'Flynn, Miss Walsh, Miss Curtin, Miss Bostock, Miss Michie, Miss Matheson, and Major Harris (Registrar). The Report of the Finance Committee for the previous half-year was adopted and the recommendations approved.

The report of the Rules Committee was then considered. The principal point concerned the fixing of a minimum standard for the admission of "existing nurses." The Council having obtained legal opinion that it is empowered to adopt one year's hospital training, it was agreed unanimously that this standard be imposed in the Rules subject to the qualification that those nurses who have been employed in Poor-law institutions, with the approval of the Local Government Board, as "qualified" nurses within the meaning of the Board's General Order of July 5, 1901, would be eligible for admission. Further points dealt with in the Draft Rules were: (1) The question of the country of registration. It was unanimously resolved that the "country of training" should determine the "country of first registration." (2) Parts VI. and VII. dealing with admission of nurses registered in Great Britain and with removal from the Register. These were referred back to the Rules Committee for redrafting, subject to certain general directions. (3) The position of the Council under the Government of Ireland Act. It was decided to recommend that there should be only one Council for all Ireland.

Parts I., II., III., and IV. of the Rules were ratified and sealed, and directions given that they be submitted to the Chief Secretary for formal approval.

